# B-waves in noninvasive capacitance signal correlate with B-waves in ICP

**DOI:** 10.1007/s00701-025-06461-3

**Published:** 2025-03-06

**Authors:** Andreas Spiegelberg, Andrea Boraschi, Ramy Amirah, Katharina Wolf, Mukesch Shah, Laura Krismer, Jürgen Beck, Vartan Kurtcuoglu

**Affiliations:** 1 The Interface Group, Department of Physiology, University of Zurich, Winterthurerstrasse 190, CH-8057 Zurich, Switzerland; 2 Department of Neurosurgery, Medical Center, University of Freiburg, Freiburg, Germany; 3 Zurich Center for Integrative Human Physiology, University of Zurich, Zurich, Switzerland; 4 Neuroscience Center Zurich, University of Zurich, Zurich, Switzerland

**Keywords:** B-waves, Slow vasogenic waves, Noninvasive measurement, Intracranial pressure, Craniospinal compliance, Normal pressure hydrocephalus

## Abstract

**Background:**

Analysis of B-waves in overnight intracranial pressure (ICP) recordings used to be an important element in the diagnosis of normal pressure hydrocephalus (NPH). Here, we tested the hypothesis that equivalents to B-waves can be detected and quantified in a noninvasively measured electric capacitance signal termed W.

**Methods:**

We measured ICP and W in a cohort of 15 patients with suspected diagnosis of NPH or spontaneous intracranial hypotension during infusion testing, identifying B-waves in both signals by wave-template matching in the time domain.

**Results:**

We found very strong correlation between the duration of B-waves in ICP and W (R^2^ = 0.86, *p* < 10^–6^), and weak correlation between the average B-wave amplitudes in ICP and W (R = 0.34, p = 0.02).

**Conclusions:**

The concurrent presence of B-waves in the signals suggests that vasogenic activity of cerebral autoregulation is reflected in W. The weaker correlation of amplitudes may be attributed to W being an indirect measure of cranial volume composition, whereas ICP is a measure of pressure, with the two linked by the non-linear craniospinal pressure-volume relation that varies between patients. Analysis of the noninvasively acquired W signal should be evaluated as a triage tool for patients with NPH and other disorders characterized by reduced compliance.

## Introduction

The analysis of B-waves in the intracranial pressure (ICP) signal used to be a mainstay in the diagnostic chain for normal pressure hydrocephalus (NPH) [1, 14, 30]. B-waves, also known as slow vasogenic waves, have a periodicity of 0.33 to 3 cycles per minute and reflect vasogenic activity of cerebral autoregulation [26]. B-waves of low amplitude are observed in healthy individuals [16, 18]. Stronger and more frequently occurring B-waves are considered to be indicative of reduced craniospinal compliance and predictors for the response to cerebrospinal fluid (CSF) shunting [22, 23, 31]. In addition, an association between B-waves and sleep apnea has been recently observed [25].

Overnight recording of ICP is the preferred way for identifying and quantifying B-waves, as they occur predominantly during REM and N2 phases of sleep [12, 35]. This practice has been largely rendered obsolete by lumbar tap testing and external lumbar drainage (ELD), which are less invasive than intraparenchymal or intraventricular ICP measurement and favored in the clinical management of idiopathic NPH [19]. However, both methods are still invasive, painful [8], and carry the risk of complications [13, 34]. In addition, ELD is labor intensive, costly, and requires hospitalization [21]. These factors have contributed to the limited adoption of lumbar tap testing and ELD.

The need for obtaining information on the craniospinal pressure-volume state noninvasively and inexpensively has motivated the development of new technologies [2, 4, 15, 27, 32]. One of these relies on the measurement of the head’s electric capacitance [28]: Two electrically isolated capacitor electrodes on the scalp form, together with an electronic device, an oscillator circuit that ultimately yields an electric signal termed W. The W signal contains, among other features, characteristic oscillations produced by cardiorespiratory action. These originate in changes of intracranial CSF and blood volumes that occur with each cardiac and respiratory cycle, causing periodic variations in the dielectric properties of the head [5, 6, 11, 28].

Based on the understanding that B-waves are caused by periodic cerebrovascular volume changes, and that the W signal reflects variations in intracranial volume composition, we formulated the following hypothesis: If B-waves are observable in ICP, then there will be correlated equivalents to them in W. To test this hypothesis, we simultaneously acquired both W and ICP in 15 patients who all underwent infusion testing, identified B-waves in both signals, and performed correlation analyses.

## Materials and methods

### Patients and procedures

Seventeen consecutive patients with scheduled infusion testing for NPH or spontaneous intracranial hypotension (SIH) were considered for inclusion in the study.Two patients had to be excluded due to technical obstacles to record the W signal (one case of electrodes coming loose unnoticed, one of a patient not being able to refrain from constant head movement). The remaining 15 (8 female, age 69.5 ± 16.1 years, 10 suspected NPH, 5 suspected SIH) were included.

Patients were placed in lateral recumbent fetal position. An 18G Sprotte needle (Pajunk GmbH, Geisingen, Germany, REF: 321151−30F) or a 5F lumbar catheter (Spiegelberg GmbH & Co. KG, Hamburg, Germany, REF: ELD33.010.02) were placed for access to the lumbar CSF space. Patients who received a lumbar catheter were then positioned in the supine position. Those with lumbar needle were left in the lateral position. All patients underwent infusion testing at an infusion rate of 1.5 mL/min or 2 mL/min as part of the standard diagnostic workup. The infusion was stopped after 50 mL had been infused or when a CSF pressure of 50 mmHg was reached or if the patient developed a strong headache. In patients with lumbar needle, 30 mL of CSF was withdrawn and the lumbar needle removed following the infusion test once CSF pressure had normalized. In patients with lumbar catheter, CSF was drained for three days as required for the routine diagnostic assessment. During the infusion test, patients were advised to keep their eyes closed, not to move, and not to speak.

Intracranial pressure was acquired through the lumbar access, a height-adjusted pressure transducer (CODAN PVB Critical Care GmbH, Forstinning, Germany, REF: 74.4398), and a pressure monitor (Raumedic AG, Helmbrechts, Germany, REF: 094 474–002). Continuous blood pressure and ECG were acquired with a Finapres*® NOVA* (Finapres Medical Systems, Enschede, The Netherlands). W was acquired using a Cephalotec PEM 1 device with modified frequency response characteristics to allow observations in the B-wave spectrum and outfitted with PEM 2 electrodes (Cephalotec, Horgen, Switzerland). The electrodes were placed symmetrically on the patient’s forehead in areas corresponding to the location of the F3, F7 and F4, F8 electrodes of a 10–20 electroencephalogram setup [7]. All signals were sampled at a rate of 100 samples per second, transmitted to a laptop computer via USB and recorded using ICM + software (University of Cambridge Enterprise, REF: ICM + 9.1.9 17). Patient symptoms, final diagnoses, therapies, and outcomes were not considered for this study, as it is purely aimed at investigating associations between ICP and W. Conversely, W was not used for diagnostic purposes.

### Identification of B-waves

ICP and W signal assessments were performed offline in Matlab (The MathWorks, Inc., Natick, MA, USA, Release: 2022b) using an automated algorithm sequence. Therein, signals were screened for implausibly high values and gradients that can be caused by external factors such as sudden body movement. Corresponding artifact-containing periods were excluded from further analysis. For visual inspection, a multi-bandpass filter was applied (4^th^ order Butterworth, pass bands: 0.5-5 Hz and 0.00555 Hz-0.05 Hz). This allowed both B-waves and pulse waves to be discerned by eye.

To identify and quantify the presence of B-waves, we used a cross-correlation technique as described previously [29]. Briefly, signals were compared to 85 sinusoidal templates containing two full B-wave periods each. These templates are representative of expected B-wave shapes and cover the full B-wave frequency range. Time windows within which the cross-correlation was found to be above a predetermined threshold were classified as B-wave trains, and their amplitudes determined (${A}_{B}^{ICP}$ and ${A}_{B}^{W}$ for ICP and W, respectively). For the ICP signal, the threshold determined in [29] was used. For the W signal, a threshold level that yielded comparable values for aggregate sensitivity and specificity was chosen (67.1% and 66.1%, respectively; see Appendix for derivation). The occurrence of B-waves in ICP was not used for diagnostic purposes.

### Quantification of B-waves

For each patient, the total duration of B-wave occurrence in ICP (denoted as ${T}_{B}^{ICP}$) and W (denoted as ${T}_{B}^{W}$) was determined as the sum of the durations of all not overlapping B-wave trains in the respective signal (see appendix, Fig. 4). With the total duration of concurrently artifact-free time denoted as $T$, the time fraction of B-wave occurrence is defined as follows:

$$\begin{array}{cc}\mathrm F_{\mathrm B}^{\mathrm{ICP}}=\frac{\mathrm T_{\mathrm B}^{\mathrm{ICP}}}{\mathrm T}\qquad\quad&\mathrm F_{\mathrm B}^{\mathrm W}=\frac{\mathrm T_{\mathrm B}^{\mathrm W}}{\mathrm T}.\end{array}$$

The mean amplitude of the B-waves (denoted as ${\overline{A} }_{B}^{ICP}$ and ${\overline{A} }_{B}^{W}$ for the respective signal) was determined as the root mean square of the individual B-wave amplitudes.

### Comparison of B-wave metrics in ICP and W signals

Pearson’s linear correlation was used to assess the relationships between the two signals in terms of mean B-wave amplitudes, as well as total durations and time fractions containing B-waves. The correlation coefficients (R^2^) and corresponding p-values were calculated to determine the strength and significance of these relationships.

## Results

**Fig. 1:**
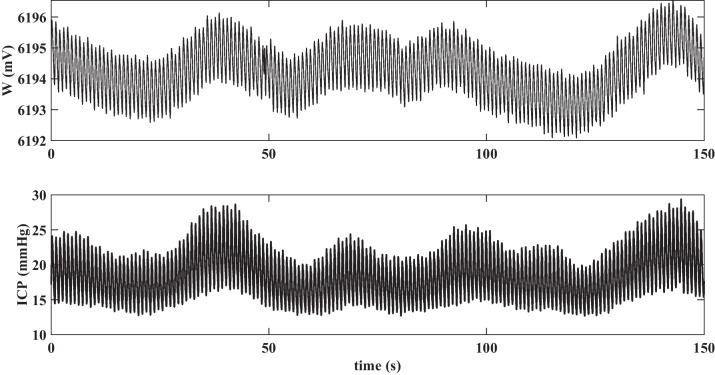
Illustrative B-wave-containing segments of W and ICP at the ICP plateau phase of the infusion test in a patient (male, 82 years) with suspected NPH

To assess whether B-waves were present in W, we first performed visual analysis of the bandpass filtered signals. In 14 of the 15 datasets, B-waves could be observed in the artifact-free parts of ICP and of W. This was not unexpected, since fluid infusion into the CSF space reduces craniospinal compliance, increasing the likelihood of B-wave occurrence. Figure 1 shows exemplary W and ICP tracings at the ICP plateau phase of the infusion test in an 82-year-old male patient under evaluation for suspected normal pressure hydrocephalus. B-waves are clearly visible in both ICP and W.

**Fig. 2:**
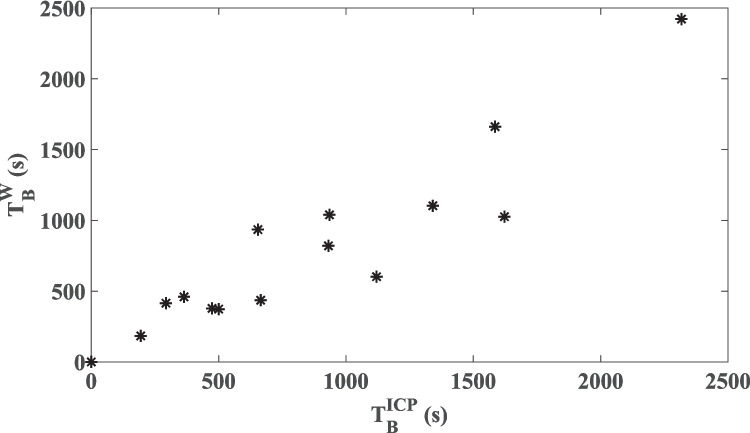
Plot of total duration of B-waves in W $\left({T}_{B}^{W}\right)$ versus the total duration of B-waves in ICP (${T}_{B}^{ICP}$). Each point corresponds to one patient dataset. A very strong correlation between the two signals with respect to total B-wave duration was found (R^2^ = 0.86, *p* < 10^–6^)

To assess the correlation between the ICP and W signals with respect to the cumulative duration of time periods with the presence of B-waves, we performed a linear correlation analysis. Each patient dataset constituted one datapoint, characterized by the total duration of B-waves in ICP $\left({T}_{B}^{ICP}\right)$ and W $\left({T}_{B}^{W}\right)$. As illustrated in Fig. 2, there is a very strong (R^2^ = 0.86) significant (*p* < 10^–6^) correlation between W and ICP for the occurrence duration of B-waves.

**Fig. 3:**
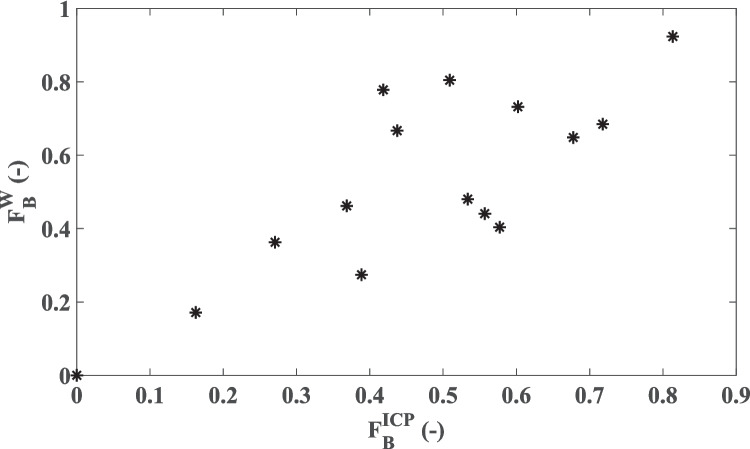
Plot of time fraction of B-wave occurrence in W $\left({F}_{B}^{W}\right)$ versus the time fraction of B-wave occurrence in ICP $\left({F}_{B}^{ICP}\right)$. Each point corresponds to one patient dataset. A moderate correlation between the two signals with respect to B-wave time fraction was found (R^2^ = 0.64, *p* = 10^–4^)

We further analyzed the correlation between the two signals in terms of time fraction of B-wave occurrence (${F}_{B}^{ICP}$ and ${F}_{B}^{W}$). In this analysis, we found a moderate (R^2^ = 0.64) significant (*p* = 10^–4^) correlation as shown in Fig. 3. Finally, we assessed the correlation between mean B-wave amplitudes in ICP and W (${\overline{A} }_{B}^{ICP}$ and ${\overline{A} }_{B}^{W}$), observing a weak (R^2^ = 0.34) significant (*p* = 0.02) correlation.

## Discussion

The dielectric properties of the head change in synchrony with cardiovascular and respiratory cycles. This temporal variation can be measured, producing the signal W. We hypothesized that W contains equivalents to B-waves when B-waves are present in the ICP. Our data confirm this hypothesis.

Visual inspection and manual comparison of the time course of simultaneously recorded W and ICP signals showed the presence of concurrently occurring oscillations in the B-wave frequency band of 0.0055 to 0.05 Hz. This indicates that the vasogenic activity of cerebral autoregulation is reflected in the noninvasively acquired signal W.

The shapes of B-wave equivalents observed in W were similar to those of B-waves in the ICP signal. This allowed us to employ a previously developed technique based on wave template matching in the time domain to automatically identify and characterize B-waves both in ICP and in W. We found a very strong linear correlation between the total duration of automatically detected B-waves in ICP and W. In other words, recordings that had many B-waves in ICP were also found to have many in the W signal.

We found a weak linear correlation between the mean B-wave amplitudes in ICP and W. The fact that this correlation is weaker than the correlation between B-wave durations in ICP and W is not surprising. While the W signal reflects volume changes, ICP represents a pressure signal. Intracranial pressure and craniospinal volume are interconnected through a non-linear pressure-volume relation. Therefore, the relation between W and ICP – and, by extension, the B-wave amplitudes in these signals – is likely to be non-linear as well. Additionally, since it is known that the pressure-volume relation can vary between patients, the relation between B-wave amplitudes in W and ICP is also likely to differ.

**Fig. 4:**
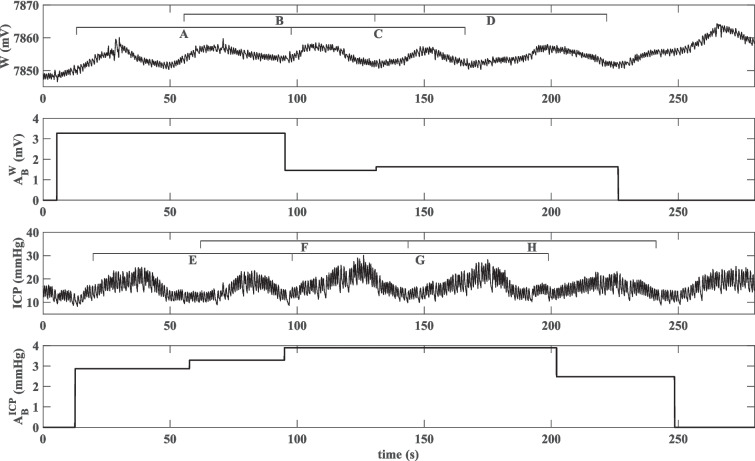
Illustrative B-wave-containing segments of ICP and W (top and third graph, respectively) at the ICP plateau phase of the infusion test in a patient (male, 82 years) with suspected NPH. Brackets A to D and E to H mark identified trains of B-waves (two full periods each) in ICP and W, respectively. The B-wave amplitude courses ${A}_{B}^{ICP}$ and ${A}_{B}^{W}$ shown in the second and bottom graphs were determined based on the amplitude of each wave train. In cases of overlapping trains, the amplitude value of the highest train was selected. Time-averaging of these amplitude courses yields mean B-wave amplitudes ${\overline{A} }_{B}^{ICP}$ and ${\overline{A} }_{B}^{W}$ of 3.22 mmHg and 2.27 mV, respectively

In our analysis, we compared B-waves in the signals to template wave trains consisting of two full periods rather than individual B-waves due to the structure of the identification algorithm. However, analyzing individual B-waves could offer further insights, such as enabling automated analysis of phase shifts that can be discerned by eye (see Figs. 1 and 4). Improvements in automated detection might also reveal closer correspondence between B-waves in ICP and W.

Noninvasively obtained cranial signals other than W have also been shown to contain oscillations in the B-wave frequency range. They have been observed with transcranial Doppler flowmetry (TCD) [9, 10, 16] and near-infrared spectroscopy (NIRS) [3, 20, 33]. However, their clinical application for B-wave analysis has not yet been established. One reason for this is that the measured oscillations in the B-wave frequency range in TCD and NIRS are thought to reflect fluctuations in cerebral blood flow and oxygenation, but without the compliance-dependent amplification observed in ICP.

The high linear correlation between the duration of B-waves in W and ICP suggests that the B-waves in the W signal may have clinical utility for the triage of patients suspected of NPH or other pathologies characterized by chronically reduced intracranial compliance, such as idiopathic intracranial hypertension. Conversely, the quantification of B-waves is less likely to be of value for the study of pathologies characterized by rapid changes in craniospinal compliance (such as in the acute phases of traumatic brain injury and intracranial hemorrhage), given the time it takes for B-waves to develop.

Longer term monitoring of W could be of interest for assessing disease evolution or for evaluating the effect of therapeutic measures. The advantage of using W rather than ICP for B-wave analysis lies in the noninvasive acquisition of the capacitive signal. This advantage would be forfeited by utilizing an invasive method (such as infusion testing) to provoke B-waves. Since B-waves occur naturally in REM and N2 phases of sleep [12, 35], overnight acquisition of W could be used as the basis for B-wave analysis. Growing interest surrounds the potential links between B-waves, sleep apnea, and cyclic alternating patterns observed in electroencephalography [17, 24, 25], In this context, monitoring of W could facilitate studies in larger patient cohorts where invasive ICP measurements may be ethically or practically unfeasible.

## Conclusions

We found B-waves in the noninvasively measured capacitive W signal to very strongly correlate with B-waves in ICP. Under the premise that more frequent occurrence of B-waves is indicative of impaired craniospinal compliance, the acquisition and analysis of W as a triage tool for patients with suspected reduced compliance should be further evaluated. 

## Figures and Tables

**Fig. 5:**
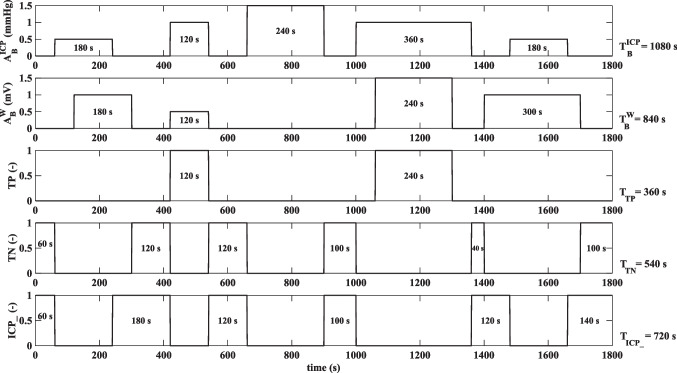
Derivation of the mean amplitudes of the B-waves in ICP $\left({\overline{A} }_{B}^{ICP}\right)$ and W $\left({\overline{A} }_{B}^{W}\right)$, the total duration of B-wave occurrence in ICP $\left({T}_{B}^{ICP}\right)$ and W $\left({T}_{B}^{W}\right)$, the total true positive duration (${T}_{TP}$), the total true negative duration (${T}_{TN}$), and the total time without presence of B-waves (${T}_{ICP\_}$). The top two graphs show the time course of B-wave amplitudes ${A}_{B}^{ICP}$ and ${A}_{B}^{W}$ identified in hypothetical ICP and W signals, respectively. Time segments where both are simultaneously > 0 are considered true positive durations (third graph, $TP=1$). When both are equal to zero, this is considered a true negative duration (fourth graph, $TN=1$). The last graph marks time segments without the occurrence of B-waves in ICP (${ICP}_{-}=1$ in the absence of B-waves). The time course of ${A}_{B}^{ICP}$ reveals a total of 5 B-wave segments with respective amplitudes of 0.5, 1, 1.5, 1, and 0.5 mV, and durations of 180, 120, 240, 360, and 180 s. ${\overline{A} }_{B}^{ICP}$ is then calculated as the duration-weighted average of the individual segments’ amplitudes (here yielding 0.94 mmHg; corresponding process for ${\overline{A} }_{B}^{W}$, yielding 1.07 mV). ${T}_{B}^{ICP}$ is calculated as the sum of the B-wave segment durations (here 1080 s). The other total durations are also calculated by summation (${T}_{B}^{W}=840\;s$, ${T}_{TP}=640\;s$, ${T}_{TN}=540\;s$, ${T}_{ICP\_}=720\;s$)

## Data Availability

The data that support the findings of this study are available from the corresponding author upon reasonable request after approval by the ethics committee.

## Appendix

### Quantification of B-wave-containing periods

B-wave amplitude courses ${A}_{B}^{ICP}$ and ${A}_{B}^{W}$ were determined based on the amplitude of each wave train (Fig. 4). In cases of overlapping trains, the amplitude value of the highest train was selected. Time-averaging of these amplitude courses yielded the mean B-wave amplitudes ${\overline{A} }_{B}^{ICP}$ and ${\overline{A} }_{B}^{W}$, respectively.

### Calculation of sensitivity and specificity

Time periods containing B-waves in the W signal were labeled as true positive if the ICP signal also contained B-waves (see Fig. 5). If there was no simultaneous occurrence in ICP, they were labeled false positive. Conversely, time periods with no B-waves in the W signal were labeled as true negative if the ICP signal also had none, and false negative if the ICP signal did contain B-waves. The sum of all time periods with true positive label yielded the total true positive duration $\left({T}_{TP}\right)$, the sum of those with true negative the total true negative duration $\left({T}_{TN}\right)$. The total time without presence of B-waves in the artifact-free ICP signal was denoted ${T}_{ICP\_}$. For each patient, sensitivity $\left(Se\right)$ and specificity $\left(Sp\right)$ of B-waves in the W signal as indicators for the occurrence of B-Waves in ICP were then calculated as follows:

$$\begin{array}{cc}Se=\frac{{T}_{TP}}{{T}_{B}^{ICP}}\qquad\quad& Sp=\frac{{T}_{TN}}{{T}_{ICP\_}}\end{array}$$

Pooled sensitivity $\left(\overline{Se }\right)$ and pooled specificity $\left(\overline{Sp }\right)$ were determined by considering the sum of true positive, true negative, B-wave containing and B-wave free times of all patients:

$$\begin{array}{cc}\overline{Se }=\frac{\sum {T}_{TP}}{\sum {T}_{B}^{ICP}}\qquad\quad& \overline{Sp }=\frac{\sum {T}_{TN}}{\sum {T}_{ICP\_}}\end{array}$$
